# Intramuscular Placement of Birth Control Device: A Multimodality Case and Review

**DOI:** 10.7759/cureus.3835

**Published:** 2019-01-07

**Authors:** Travis Z Johnson, Trevor Annis, Anne Kennedy, Patrick Kobes, Megan Mills

**Affiliations:** 1 Radiology, University of Utah School of Medicine, Salt Lake City, USA; 2 Radiology, University of Utah Hospital, Salt Lake City, USA

**Keywords:** nexplanon, contraceptive, birth control, imaging, implantable, computed tomography, magnetic resonance imaging, ultrasound

## Abstract

Implantable forms of birth control have been used as a means for convenient and effective medication delivery. Nexplanon® (Merck Sharp & Dohme B.V., Haarlem, the Netherlands) is an implantable birth control device placed in the subcutaneous tissue of the upper medial arm during an in-office procedure. The location of the device is usually confirmed by palpation during a physical exam. In cases where the device is non-palpable, imaging may aid in localization. Implantable birth control devices have a characteristic radiologic appearance, and the location of the device can be confirmed by utilizing ultrasound and/or radiography. Occasionally, sonographic and radiographic studies may have equivocal results necessitating advanced imaging with computed tomography (CT) and/or magnetic resonance imaging (MRI). Subcutaneous location of the device is essential for efficacy and safety. An intramuscular position of the device is inappropriate and may lead to insufficient medication delivery and/or injury to the deep soft tissues. This case reviews the imaging features of a Nexplanon device which had been inappropriately placed into the muscle.

## Introduction

The creators of the Nexplanon® (Merck Sharp & Dohme B.V., Haarlem, the Netherlands) device describe it as a safe and convenient form of implantable birth control [1]. The device, which is about the size and shape of a matchstick, is implanted in the subcutaneous tissue of the upper medial arm during an in-office procedure. It is effective at preventing pregnancy for up to four years.

In this case, a slim (21.1 kg/m^2^) and athletic 30-year-old woman presented for removal of a Nexplanon birth control device, which had been placed in the left medial arm after her first pregnancy three years prior. She noted that the device had been an effective contraceptive but had never been palpable. In-office localization was attempted but was unsuccessful. The patient was then referred to radiology for ultrasound localization.

## Case presentation

The location of an implantable birth control device is usually confirmed by palpation during a physical examination. In cases where physical exam fails, Gurel et al. described the characteristic radiologic appearance that usually allows for accurate localization via ultrasound and/or radiography [2].

**Figure 1:**
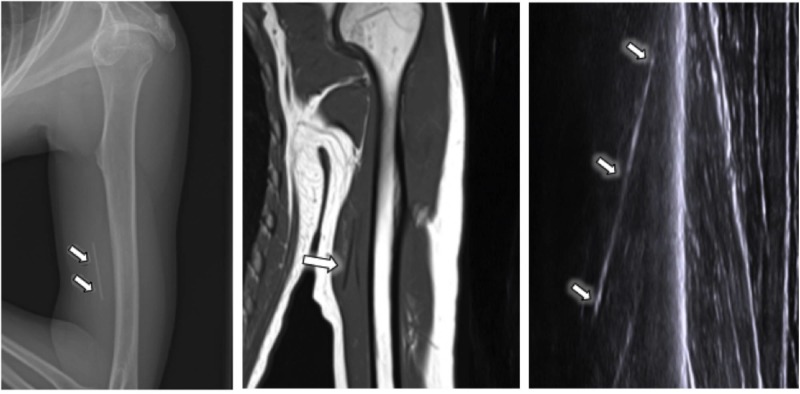
Radiograph, MRI, and an ultrasound image of the left upper extremity A radiograph, MRI, and an ultrasound image of the left upper extremity show an implantable birth control device (arrows) inappropriately located within the triceps musculature. Note the geographic appearance of the 4 cm device completely surrounded by muscle tissue, the lack of acoustic shadowing, and the similarity to other fascial planes on ultrasound. MRI: magnetic resonance imaging

In this case, an obliquely-oriented linear structure was visible sonographically within the deep soft tissues of the medial arm; however, despite imaging by two experienced operators and the use of different sonographic parameters, the location could not be verified in cross-section. In particular, the typical distal acoustic shadowing seen in cross-section was not demonstrable due to its location in the muscle. The concern was that the non-shadowing, linear sonographic echo could represent a fascial plane rather than an implanted device. Radiographs confirmed that the implant was indeed present but was positioned deep within the musculature of the medial arm. Its proximity to the humerus and deep muscular location may have contributed to the difficulty in confirming its location in two planes sonographically, which is essential for surgical planning. Thus, in an effort to avoid injury to the musculature and adjacent deep tissues during the “blind” surgical removal, magnetic resonance imaging (MRI) was performed. The MRI localized the device to the medial head of the triceps without the involvement of the vascular structures or other muscle compartments (Figure 1). MRI interpretation was facilitated by a review of the radiographs and the video clips obtained during the ultrasound examination. Following confident localization, surgical removal of the device was performed uneventfully.

## Discussion

Ultrasonography with high-frequency transducers is recommended as the first-line method for localization of a non-palpable birth control implants. Salsamendi et al. described how imaging can be used to localize a device and may serve as an adjunct to evaluate for other complications, such as device discontinuity [3]. Little is known about the clinical consequences of fractured implants and about the identification of the fractured or misplaced device. Crouthamel et al. described the rarity of fractured hormonal implants through a crowd-sourced series of 54 cases [4]. Out of 42 responses, mechanisms of implant fracture were found to be patient manipulation (23%, n = 12), unintentional trauma (11%, n = 6), interpersonal violence (8%, n = 4), lifting/carrying (6%, n = 3), fracture with removal (6%, n = 3), and unknown (47%, n = 25). The time interval between placement and fracture was less than two years for 63% (n = 34) of cases. Understanding the mechanism behind hormonal implant fracture and the follow-up care needed is essential in treating patients with these issues. Thurmond et al. stated that implanted contraceptive capsules occasionally cannot be removed by means of palpation with local dissection, and imaging guidance is necessary [5]. Her case described the need of imaging to localize and remove a device from the arm of a patient. The typical appearance of a Nexplanon rod is usually seen on longitudinal and transverse ultrasound scanning of the arm. Its diameter and superficial, highly echogenic, linear structure produce strong posterior acoustic shadows. Shadowing is best featured on transverse images.

With proper subcutaneous tissue implantation of a Nexplanon device, palpation and ultrasound are sufficient for its safe in-office removal. However, in cases where the device is non-palpable, further imaging with radiography, computed tomography (CT), or MRI can assist in localization and planning for safe removal.

Biskamp et al. described the rarity of intramuscular placement and the necessity for operative removal of the device in these circumstances [6]. Occasionally, sonographic and radiographic studies may have equivocal results, necessitating advanced imaging with a CT and/or MRI. Subcutaneous placement of the device is essential for efficacy and safety. Intramuscular implantation is inappropriate and may lead to insufficient medication delivery and/or injury to the deep soft tissues.

## Conclusions

Intramuscular placement of an implantable contraceptive device is uncommon. Multiple imaging modalities may be needed for accurate anatomic localization, which is a prerequisite to safe removal of the inappropriately placed device.
